# Repurposing Lesogaberan to Promote Human Islet Cell Survival and *β*-Cell Replication

**DOI:** 10.1155/2017/6403539

**Published:** 2017-09-05

**Authors:** Jide Tian, Hoa Dang, Angela Hu, Willem Xu, Daniel L. Kaufman

**Affiliations:** Department of Molecular and Medical Pharmacology, University of California, Los Angeles, CA, USA

## Abstract

The activation of *β*-cell's A- and B-type gamma-aminobutyric acid receptors (GABA_A_-Rs and GABA_B_-Rs) can promote their survival and replication, and the activation of *α*-cell GABA_A_-Rs promotes their conversion into *β*-cells. However, GABA and the most clinically applicable GABA-R ligands may be suboptimal for the long-term treatment of diabetes due to their pharmacological properties or potential side-effects on the central nervous system (CNS). Lesogaberan (AZD3355) is a peripherally restricted high-affinity GABA_B_-R-specific agonist, originally developed for the treatment of gastroesophageal reflux disease (GERD) that appears to be safe for human use. This study tested the hypothesis that lesogaberan could be repurposed to promote human islet cell survival and *β*-cell replication. Treatment with lesogaberan significantly enhanced replication of human islet cells *in vitro*, which was abrogated by a GABA_B_-R antagonist. Immunohistochemical analysis of human islets that were grafted into immune-deficient mice revealed that oral treatment with lesogaberan promoted human *β*-cell replication and islet cell survival *in vivo* as effectively as GABA (which activates both GABA_A_-Rs and GABA_B_-Rs), perhaps because of its more favorable pharmacokinetics. Lesogaberan may be a promising drug candidate for clinical studies of diabetes intervention and islet transplantation.

## 1. Introduction

A major goal in diabetes research is to develop agents that can safely promote human *β*-cell survival and replication. Most mitogens and growth factors that have been shown to promote rodent *β*-cell replication fail to promote human *β*-cell replication (reviewed in [1, 2]). *β*-Cells have been long known to express the GABA synthetic enzyme glutamic acid decarboxylase (GAD), as well as GABA_A_-Rs and GABA_B_-Rs [3–7]. Although GABA_A_-Rs and GABA_B_-Rs share GABA as an agonist, these receptors are encoded by distinct gene families and their activation induces different pathways; GABA_A_-Rs are fast-acting chloride channels and GABA_B_-Rs are slow-acting G-protein-coupled receptors [8, 9]. Recently, GABA administration has been shown to protect rodent and human *β*-cells from apoptosis and to promote their replication both *in vitro* and *in vivo* [10–14]. This response is mediated by both GABA_A_-R and GABA_B_-Rs [10–12, 14, 15]. GABA-mediated enhancement of *β*-cell replication did not attenuate after five weeks of GABA treatment and led to an eventual increase in *β*-cell mass in a nonautoimmune context [14]. Notably, GABA treatment enhanced *β*-cell replication and survival in newly diabetic NOD mice [11, 16, 17], indicating that GABA-R activation can be beneficial in an autoimmune context even when little *β*-cell mass remains.

*β*-Cells express GABA_A_-Rs and GABA_B_-Rs [3, 4, 7, 18, 19]. *α*-Cells express GABA_A_-Rs but may not express functional GABA_B_-Rs, while PCR detects GABA_B_-R transcripts in isolated *α*-cells (but not the *α*-cell line *α*-TC9), a GABA_B_-R agonist failed to modulate any of the tested *α*-cell functions [3, 18–21]. We are unaware of any evidence of functional GABA_B_-Rs on *δ* or PP islet cells. Very recently, long-term treatment with antimalarial drugs that target gephyrin (a protein that participates in GABA_A_-R transport to the membrane), or treatment with GABA, was shown to promote islet *α*-cell transdifferentiation into *β*-cells [22, 23]. This conversion appears to be mediated by GABA_A_-Rs [22, 23] and not the GABA_B_-Rs that are the focus of this study.

GABA appears safe for human consumption [24] and its inability to pass through the blood-brain barrier (BBB) avoids potential CNS effects. GABA, however, has a relatively low affinity for its receptors, a fast off-rate (presumably so that neurons can quickly respond to the next stimulus), and a short half-life (about 20 minutes in blood [25]) and therefore may be pharmacologically suboptimal. In the clinic, there are many BBB-permeable drugs that can modulate neuronal GABA-Rs and ameliorate CNS disorders such as seizures, anxiety, and insomnia. Their CNS effects, however, raise concerns about their long-term use for the treatment of diabetes. It would be ideal if peripherally restricted GABA-R agonists that were safe for human use could be repurposed for treating diabetes.

Lesogaberan (AZD3355) is a peripherally restricted GABA_B_-R-specific agonist that was developed for the treatment of GERD [26–28]. It has an EC_50_ of 9 nM (compared to GABA's EC_50_ of 160 nM) and a *K_i_* of 5 nM (versus 110 nM for GABA) for GABA_B_-Rs [26] and a half-life of about 11 hours in peripheral blood [29, 30]. While treatment of GERD patients with lesogaberan (oral 60–240 mg twice daily) for up to 28 days in several phase IIb clinical trials did not result in sufficient beneficial effects, there were no treatment-related serious adverse events, suggesting that lesogaberan may be safe for human use [27, 29, 31]. Here, we tested the potential of repurposing lesogaberan to promote human islet cell survival and *β*-cell replication. Our findings suggest that targeting GABA_B_-Rs can promote human *β*-cell replication and islet cell survival to a similar extent as GABA.

## 2. Materials and Methods

### 2.1. Chemicals

Lesogaberan was supplied by AstraZeneca (London, UK). The development and structure of lesogaberan have been previously described [28]. Saclofen, streptozotocin (STZ), and 5-bromo-2-deoxyuridine (BrdU) were purchased from Sigma Aldrich.

### 2.2. Islet Cell Proliferation Assay

Fresh human islets were obtained from the Integrated Islet Distribution Program (IIDP). The islets (50–75 IEQ/well) were treated in triplicate with, or without, the indicated dosages of lesogaberan in CMRL medium (0.1% glucose, Gibco) containing 10% human AB-type sera (MP Biomedicals, Santa Ana, USA) and 1.5 *μ*Ci/ml of ^3^H-thymidine in the presence or absence of the competitive GABA_B_-R antagonist saclofen (10^−4^ M) for 4 days. Islets cultured in medium alone served as controls. The ^3^H-thymidine uptake in individual wells was measured by *β*-counter. Data were analyzed by the proliferation index formula: CPM of experimental wells / CPM of controls.

### 2.3. Analysis of Human *β*-Cell Replication *In Vivo*

All animal experiments were approved by UCLA's Animal Research Committee. NOD/scid mice were injected intraperitoneally with STZ to induce diabetes and implanted with about 2000 human islets under their kidney capsule. The mice were randomized and treated for 12 days with plain water containing 0.8 mg/ml of BrdU as the control or water containing the same dose of BrdU and lesogaberan (0.08 mg/ml) or positive control GABA (6 mg/ml). At the end of treatment, the percentages of BrdU^+^insulin^+^ and Ki67^+^insulin^+^* β*-cells in at least 2000 islet cells of 10 fields (magnification ×400) of each islet graft were determined by immunofluorescence in a blinded manner, as our previous report [12].

### 2.4. Analysis of Human *β*-Cell Apoptosis *In Vivo*

STZ-rendered diabetic NOD/scid mice were implanted with about 2000–3000 human islets under their kidney capsule. The mice were randomized and given plain water or water containing lesogaberan (0.08 mg/ml) or positive control GABA (6 mg/ml). Forty-eight hours later, the percentages of insulin^+^* β*-cells or TUNEL^+^ apoptotic islet cells in total islet cells within the grafts of individual recipients were determined by immunofluorescence in a blinded manner, as in our previous report [12].

### 2.5. Statistical Analysis

Data are expressed as the mean ± SEM of individual groups (*n* = 4–9 mice per group) from two separate experiments. The difference among groups was analyzed by ANOVA and post hoc Fisher's least significant difference and the difference between groups was determined Student *t*-test. A *p* value of <0.05 was considered statistically significant.

## 3. Results

### 3.1. Lesogaberan Enhances Human Islet Cell Proliferation *In Vitro*

**Figure 1:**
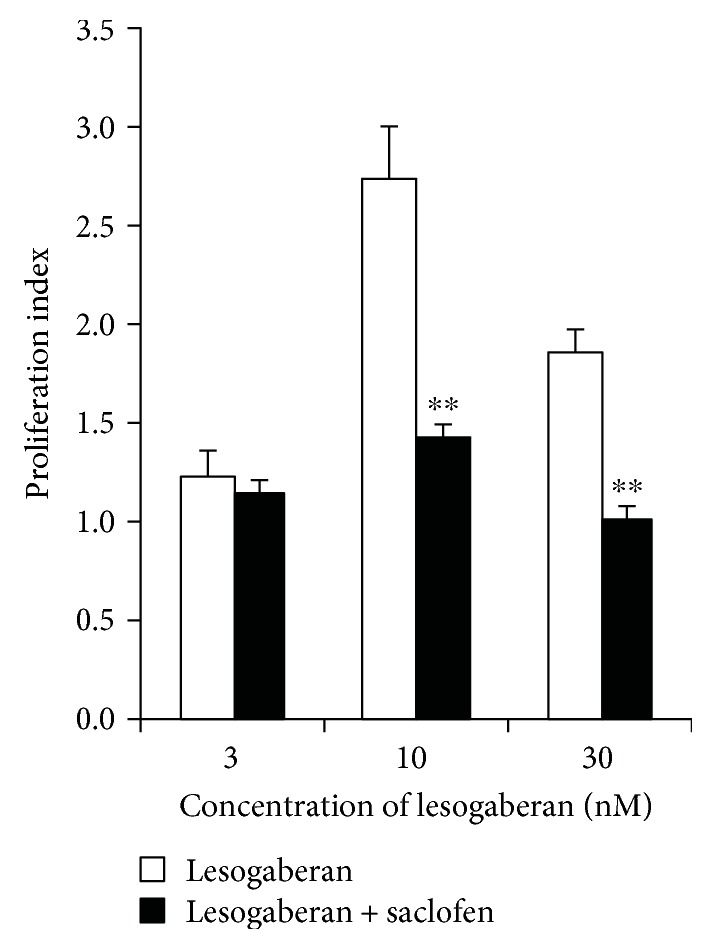
Lesogaberan enhances human islet cell proliferation *in vitro*. Human islets were treated in triplicate with, or without, the indicated dosages of lesogaberan in the presence (black bars) or absence (open bars) of saclofen (10^−4^ M) for 4 days. Data shown are the average rate of proliferation ± SEM relative to that of cultures with medium alone (designated as 1) using islets from two donors, each of which were studied in separate experiments. Treatment with saclofen alone did not affect the proliferation of human islet in our experimental system (data not shown). ^∗∗^*p* < 0.01 versus lesogaberan.

Human islets were treated with lesogaberan over a dose range in the presence, or absence, of the GABA_B_-R competitive antagonist saclofen. The effect of drug treatment on islet cell proliferation was determined by ^3^H-thymidine incorporation. We observed that lesogaberan at 3 nM had a small but nonsignificant promitotic effect, while treatment at higher dosages (10 and 30 nM) led to a 2-3-fold increase in proliferation relative to that of islets cultured in medium alone (Figure 1). Lesogaberan effects were not dose-dependent at the concentrations tested, and this may reflect its bimodal effect that was noted in some early preclinical GERD studies [26], but was not observed in subsequent clinical studies [27, 29, 31–33]. The promitotic effect of lesogaberan was blocked by saclofen (Figure 1). Hence, activation of GABA_B_-Rs enhanced human islet cell proliferation *in vitro.*

Since *β*-cells may be the only islet cell type that expresses functional GABA_B_-Rs and *α*-cell transdifferentiation would require long-term GABA_A_-R activation and would not involve ^3^H-thymidine incorporation into new DNA, the vast majority of the proliferating islet cells are likely to be *β*-cells. That contention, however, requires verification, which we pursued using quantitative immunohistochemical analysis of *β*-cells in human xenografts below.

### 3.2. Oral Lesogaberan Promotes Human *β*-Cell Replication *In Vivo*

Next, we quantitatively assessed lesogaberan's effects on human *β*-cell replication *in vivo*. We implanted ~2000 human islets under the kidney capsule of STZ-rendered diabetic NOD/scid mice. Islet recipients were provided with water containing BrdU with, or without, lesogaberan (0.08 mg/ml) or GABA (6 mg/ml) for 12 days. The lesogaberan dose was chosen based on the results of previous preclinical studies in GERD models [26, 34] and our pilot dosing studies. All islet recipients, including controls given plain water, quickly became normoglycemic after receiving the islet graft and maintained normoglycemia throughout the experimental period. There was no significant difference in their food and water consumption (~4 ml/day), as well as body weights among these groups of mice (data not shown).

**Figure 2:**
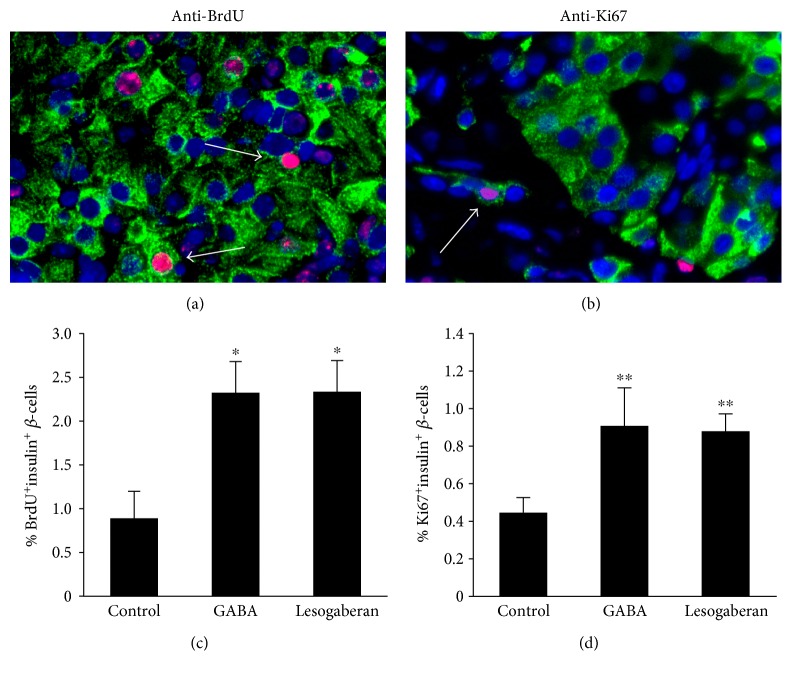
Oral lesogaberan promotes human islet *β*-cell replication in mice. Mildly hyperglycemic NOD/scid mice were transplanted with human islets under their kidney capsule. The mice were randomized and provided with water containing with BrdU, with or without GABA (6 mg/ml) or lesogaberan (0.08 mg/ml) for 12 days. The percentages of replicated *β*-cells were determined by immunofluorescent assays using anti-insulin and anti-BrdU or anti-Ki67, followed by counterstaining with DAPI. (a) Representative image of islet cells (magnification ×400) costained with anti-insulin (green) and anti-BrdU (red) (arrows). (b) Representative image of islet cells costained with anti-insulin (green) and anti-Ki67 (red) (arrows). (c) Graphical representation of the percentages of BrdU^+^insulin^+^* β*-cells and (d) Ki67^+^insulin^+^ islet cells in total insulin^+^* β*-cells. Data are mean ± SEM from two independent experiments, each using islets from a human donor that were implanted into 4–9 NOD/scid mice. The percentages of BrdU^+^insulin^+^ and Ki67^+^insulin^+^* β*-cells in at least 2000 islet cells of 10 fields of each islet graft were determined as described in Materials and Methods. ^∗^*p* < 0.05, ^∗∗^*p* < 0.01 versus the control.

Immunofluorescent staining indicated that treatment with GABA significantly enhanced human *β*-cell replication (Figure 2), similar to previous observations [14, 16]. Lesogaberan treatment also significantly increased the frequency of BrdU^+^insulin^+^ and Ki67^+^insulin^+^ islet *β*-cells relative to that in human islets from the control mice (Figure 2). There was no significant difference in the percentages of replicative *β*-cells between the lesogaberan-treated and GABA-treated groups of mice. Hence, activation of peripheral GABA_B_-Rs effectively promotes human islet *β*-cell replication *in vivo*.

### 3.3. Oral Lesogaberan Limits Human Islet Cell Apoptosis following Islet Transplantation

**Figure 3:**
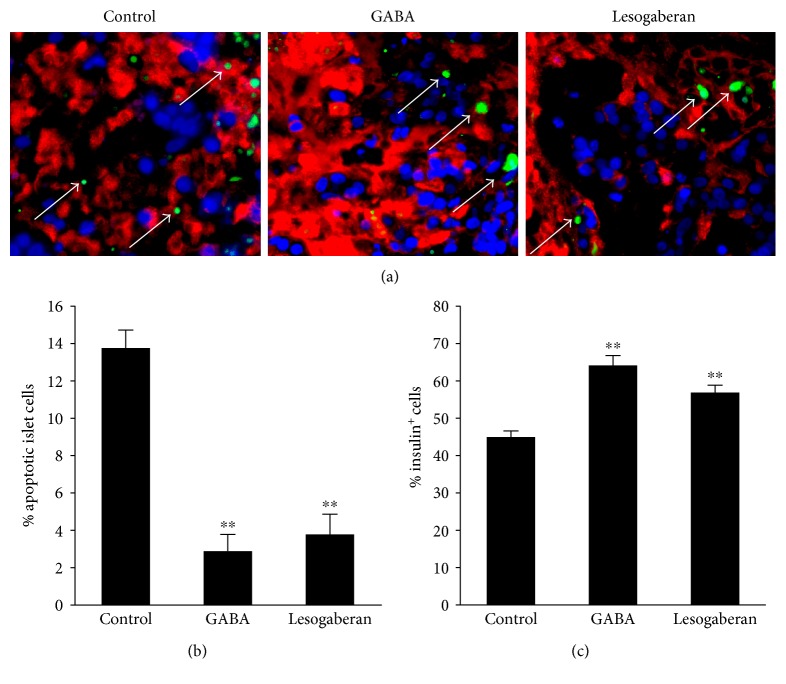
Oral lesogaberan protects human islet *β*-cells from apoptosis in islet grafts in mice. Diabetic NOD/scid mice were implanted with human islets and treated with plain water or water containing lesogaberan (0.08 mg/ml) or GABA (6 mg/ml) for 48 h. The percentages of apoptotic islet cells and islet *β*-cells in total human islet cells were determined by TUNEL assay and costaining with anti-insulin as well as DAPI. At least 2000 human islet cells in 10 fields (magnification ×400) from individual grafts were counted. Data are representative image or expressed as the mean % ± SEM for each group of mice (*n* = 5 − 7) from three separate experiments.(a) A representative image with white arrows indicating TUNEL^+^ cells (green for TUNEL^+^, red for anti-insulin^+^, and light blue for DAPI staining). (b) Percent apoptotic islet cells and (c) percent insulin^+^ islet cells. ^∗∗^*p* < 0.01 versus the control.

Following human islet transplantation, the islets are subjected to hypoxic, metabolic, and inflammatory stressors which cause the apoptosis of a large proportion of islet cells within the first few days after implantation [35, 36]. We examined the ability of lesogaberan administration to limit islet cell apoptosis in a human islet xenograft model. We implanted STZ-rendered hyperglycemic NOD/scid mice with human islets under their kidney capsule and placed them on plain water or water containing lesogaberan or positive control GABA. Two days later, their kidneys were removed and tissue sections were stained by TUNEL and anti-insulin antibodies (Figure 3). In comparison with mice given plain water, treatment with GABA significantly protected human islet cells from apoptosis, consistent with previous observations [14, 16]. Furthermore, oral feeding with lesogaberan significantly reduced the percentages of apoptotic islet cells (Figure 3(b)) and increased the frequency of insulin^+^* β*-cells in human islet grafts (Figure 3(c)). Thus, lesogaberan-mediated activation of GABA_B_-Rs limits islet cell apoptosis in transplanted human islets, consistent with previous islet cell survival studies with the prototypic GABA_B_-R-specific agonist baclofen, a drug unsuited for long-term human use [10, 12, 14].

## 4. Discussion

The modulation of GABA-Rs on *β*-cells is emerging as a new strategy to help promote human *β*-cell survival and replication in the context of T1D. Although GABA is safe for human consumption, its pharmacokinetics may be suboptimal. There are a number of available drugs that have been in wide clinical use to modulate GABA-Rs on CNS neurons; however, their CNS effects raise concerns for long-term diabetes treatment. We chose to test lesogaberan's potential for diabetes treatment based on (1) its high affinity for GABA_B_-Rs, (2) its peripheral restriction circumvents CNS effects, and (3) its apparent safety in early clinical studies.

Our initial studies showed that lesogaberan at low dosages (30 and 10 nM) increased human islet cell proliferation *in vitro* by about 2-3-fold (resp.). This promitotic effect was abrogated by a GABA_B_-R antagonist, confirming that the effect was mediated through GABA_B_-Rs. The proliferating islet cells are likely to be primarily *β*-cells because *β*-cells may be the only islet cell type that express functional GABA_B_-Rs and *α*-cell transdifferentiation requires long-term GABA_A_-R activation [22].

After implanting human islets into scid mice, lesogaberan significantly increased *β*-cell replication in the islet grafts. The level of lesogaberan-induced *β*-cell replication *in vivo* was similar to that induced by GABA at the dosages used in our model and similar to the maximum level of *β*-cell replication that takes place shortly after birth in humans [37]. The activation of *β*-cell GABA_B_-Rs causes the opening of potassium channels, release of Ca2^+^ from intracellular storage, PKA activation, and Ca^2+^-dependent activation of PI3K-Akt and CREB (reviewed in [14]). It is notable that lesogaberan promoted *β*-cell replication as effectively as GABA (which activates both GABA_B_-Rs and GABA_A_-Rs) which may be due to its superior pharmacokinetics. Our short-term treatments with lesogaberan and GABA should not have induced *α*-cell transdifferentiation (which became detectable after 2 months of GABA_A_-R activation [22, 23]), and the small increase they induced in *β*-cell replication should not alter islet size. Finally, we observed that lesogaberan treatment significantly preserved human islet cells from apoptosis in human islet grafts.

It is thought that the amount of residual *β*-cell mass following T1D onset is a major factor determining the success of interventive therapy such that even a short-term treatment with lesogaberan may help preserve residual *β*-cell mass and thereby improve the outcome of interventive therapies. Indeed, GABA treatment enhanced *β*-cell replication and survival in newly diabetic NOD mice [11, 16], demonstrating that GABA-R activation can be beneficial in an autoimmune context even when there is little residual *β*-cell mass. It is worth noting that using FDA guidelines for scaling mouse doses to the equivalent human dosage (http://www.fda.gov/downloads/Drugs/.../Guidances/UCM078932.pdf), the lesogaberan dose we used was about 2- to 7-fold lower than the doses used in phase IIb GERD clinical trials [27, 32, 33]. This suggests that it may be possible to use lower dosage lesogaberan for diabetes treatment.

Conceivably, long-term lesogaberan treatment may lead to increased *β*-cell mass in T1D patients if autoimmune responses can be sufficiently controlled. In regard to controlling *β*-cell autoimmunity, recent studies indicate that activation of GABA_B_-Rs on immune cells has anti-inflammatory effects and can ameliorate collagen-induced arthritis and contact dermatitis in mouse models [38, 39]. Therefore, lesogaberan may also have anti-inflammatory effects that help control the pathogenic autoreactive T-cell responses that mediate *β*-cell destruction in T1D. It will be of interest to further investigate how activation of GABA_B_-Rs modulates the functions of different types of immunocompetent cells, and this may be a fertile area for new anti-inflammatory drug development. Additionally, lesogaberan may be combined with other immune modulators to potentially increase therapeutic effects, as was shown by the synergistic effect of combining GABA and antigen-specific immunotherapy to reverse hyperglycemia in newly diabetic NOD mice [16].

## 5. Conclusions

We found that the GABA_B_-R agonist lesogaberan promoted human islet cell proliferation *in vitro,* as well as *β*-cell replication and islet cell survival *in vivo*, as effectively as GABA. Accordingly, GABA_B_-R agonists may provide a new drug class to help maintain residual *β*-cell mass and function after diabetes onset. Our findings also suggest that including lesogaberan in the drug regimen following clinical human islet transplantation for even a brief period may reduce *β*-cell loss due to stressors and may thereby reduce the number of islets required to achieve insulin independence. Lesogaberan's apparent safety and pharmacokinetic profile make it an excellent candidate for testing in clinical trials.
